# Expanding role for single‐pill combination drug therapy in the initial treatment of hypertension?

**DOI:** 10.1111/jch.14378

**Published:** 2021-10-17

**Authors:** Yanglong Li, Megumi Narisawa, Zhe Huang, Xiangkunm Meng, Hailong Wang, Xueying Jin, Xionghu Shen, Xian Wu Cheng

**Affiliations:** ^1^ Department of Oncology Yanbian University Hospital Yanjin Jilin PR China; ^2^ Department of Cardiology Nagoya University Graduate School of Medicine Nagoya Japan; ^3^ Department of Cardiology Yanbian University Hospital Yanjin Jilin PR China

**Keywords:** hypertension, single‐pill combination, statin, treatment

Arterial hypertension is a major health concern worldwide.^1^ High arterial blood pressure (BP) is the leading modifiable risk factor for cardiovascular disease (CVD), contributing to the greatest global burden of disease.^2^ If the presence of hypertension can be correctly diagnosed, the risk of future cardiovascular complications and events can be markedly suppressed with BP‐lowering medications and dietary and lifestyle interventions.^2^, ^3^, ^4^ However, despite the increased awareness of the importance of maintaining healthy BP values and the availability of multiple intervention options and therapies, a high percentage of hypertensive individuals fail to control their high BP and thus prevent the development of CVD. One of the major reasons for this failure is that there are major deficiencies with respect to the awareness and diagnosis of high BP, the available BP‐lowering treatments, and drug adherence; moreover, these problems persist across low‐, middle‐, and high‐income countries, emphasizing the need for widespread population‐level improvement in the understanding of hypertension.^5^ Over the last decade, BP control rates have plateaued worldwide at low levels. It was reported that the rates of BP control are 17–31% in hypertensive patients in high‐income countries, and the control rates are likely to be even poorer in low‐ and middle‐income countries.^5^, ^6^, ^7^ The 2018 European Society of Hypertension/European Society of Cardiology guidelines state that poor adherence to BP‐lowering treatment and physicians' clinical inertia (ie, lack of therapeutic action when a patient's BP is uncontrolled) are essential causes of the poor control of high BP.^8^ Overall, 43–66% of individuals with hypertension fail to adhere to their prescribed multiple antihypertension medications, and after 1 year, approx. 40% of patients with high BP stop using their initial antihypertension drugs.^9^, ^10^ In addition, about 10% of patients with hypertension forget to take their multiple pills on a daily basis.^11^ The relationship between poor treatment adherence/compliance and CVD has been widely investigated.^12^, ^13^, ^14^, ^15^, ^16^ A recent systematic review assessed whether single‐pill combination (SPC) therapy led to improved adherence, persistence, and better BP control compared to free‐equivalent combination (FEC) therapy in patients with hypertension.^7^ That review was a meta‐analysis of 18 studies comprising the cases of 1 356 188 patients with high BP, and it revealed that SPC therapy leads to improved adherence and persistence compared to FEC therapy,^7^ suggesting that SPC regimens lead to better BP control in patients with hypertension.

Over the past two decades, many attempts to enhance the clinical effectiveness of BP‐lowering SPC regimens have been made toward the goal of improving adherence to BP medications, including the use of regimens with two different types of antihypertensive drugs, eg, diuretics, β‐blockers, calcium channel blockers (CCBs), angiotensin‐converting‐enzyme inhibitors (ACEIs), angiotensin II receptor blockers (ARBs), and renin inhibitors (RIs). Current clinical therapeutic challenges with SPC regimens containing more than two antihypertensive drugs are designed to reach a target BP in order to reduce the risk of cardiovascular complications and outcomes. In this issue of the *Journal of Clinical Hypertension*, Lee and colleagues present intriguing results concerning the clinical effectiveness and safety of amlodipine/losartan‐based SPC therapies, ie, amlodipine + losartan (AL)‐, amlodipine + losartan + rosuvastatin (ALR)‐, and amlodipine + losartan + chlorthalidone (ALC)‐based treatment in patients with hypertension using real‐world, multi‐center observational databases,^1^ and they describe three important new findings: (1) AL‐based SPC treatment provided a significant BP reduction rate, (2) rosuvastatin‐combination SPC therapy provided a better reduction rate for target low‐density lipoprotein‐cholesterol (LDL‐C) compared to other SPC therapies, and (3) all three AL‐based SPC therapies showed a good safety profile and excellent drug adherence.

One of the main findings of this study is that the target BP‐lowering rate of three AL‐based SPCs over a long‐term assessment was > 90%, which is considerably higher than the average BP control rate of hypertensive patients undergoing interventions in Korea (71%) as well as those in countries, including Canada, Germany, and the U.S. (70–85%).^17^, ^18^ A lower number of pills for a patient's treatment has been associated with better drug adherence and BP reduction.^19^ In current study, the drug adherence rate for AL‐based SPCs was 91.5%, which is higher than or comparable to the rates in earlier investigations of SPCs.^20^, ^21^ Taken together with the above‐cited systematic review's^7^ finding that an SPC‐mediated improvement of drug adherence resulted in excellent BP reduction in patients with hypertension, our findings suggest the achievement of improved drug compliance due to a lowered pill burden in AL‐based SPC treatment for hypertensive patients who were already taking multiple pills.

Lee and colleagues also examined the efficacy of AL‐based SPCs on high LDL‐C levels in hypertensive patients with dyslipidemia. Because hypertensive individuals often take multiple pills, they are sometimes reluctant to take additional lipid‐lowering drugs. It was recently reported that some hypertensive patients with dyslipidemia still did not have a cholesterol‐lowering drug even though they have hypertensive complications.^22^, ^23^ A group of patients treated with an ALR regimen (62.1% started taking statins) had a significant reduction of target LDL‐C compared to an AL‐treated group (74.8% vs 89.1%, *p* < .01),^16^ suggesting that SPCs containing a statin could be an attractive treatment option to improve the lipid control in patients who are taking multiple pills by improving their drug adherence.

In addition, Lee et al. evaluated the safety of AL‐based SPC therapies, focusing on the influence of uric acid (UA) levels. Similar to common C‐reactive protein, elevated serum UA levels have been known to be one of the surrogate markers for inflammation in hypertensive patients.^24^, ^25^, ^26^ The blood UA level is often targeted in attempts to counter the risk of side effects of BP‐lowering drugs.^26^ The ARB losartan has been shown to suppress serum UA levels through the inhibition of urate transporter 1.^26^ Consistently, with the exception of the ALC group, the authors have shown that AL‐ and ALR‐based SPC therapies exerted a blood UA‐lowering effect in hypertensive patients,^16^ suggesting that the losartan‐based SPC‐mediated anti‐hyperuricemia action contributes to these therapies' safety. In contrast, a losartan‐based SPC regimen containing the diuretic chlorthalidone resulted in an increase in the patients' serum UA levels (0.2 mg/dL, *p* = .12), although the rate of new‐onset hyperuricemia was higher in the ALC group than in the AL and ALR groups (13% vs 6.9% vs 3.9%),^16^ indicating that this result might be due to the uric acid‐lowering effect of losartan in the ALC group. Further prospective studies are needed to evaluate symptomatic adverse symptoms (eg, dizziness, headache, and gastrointestinal symptoms) in AL‐ and ALR‐treated patients, as in previous studies.^27^

An important limitation of the present study is that it was a retrospective analysis of 15 538 hypertensive patients treated with AL‐based SPCs from the Observational Medical Outcomes Partnership Common Data Model database of three tertiary hospitals in Korea. Other important clinical issues that require consideration and exploration include (i) comparisons of the rates of BP reduction provided by AL‐based SPC therapies with other SPC regimens for patients with mild to moderate hypertension; (ii) comparisons of the rates of plasma LDL‐C reduction provided by ALR‐based SPC therapies with those of related SPC regimens for hypertensive patients with dyslipidemia; and (iii) clarifying whether two SPC regimens exert beneficial effects on cardiovascular complications and outcomes in patients with resistant hypertension over long‐term follow‐up periods.

In conclusion, in light of the findings suggesting ALR‐ and AL‐based SPC regimens as a new pharmacological option for the treatment of hypertensive patients with or without dyslipidemia, future studies of the underlying mechanisms can be expected to shed light on how the AL‐ and ALR‐mediated lowering of high BP produces cardiovascular benefits in hypertensive patients and animal models with and without complications (eg, diabetes mellitus, stroke, acute myocardial infarction, occlusive lower extremity arterial disease, and tumors). Together, the past and present findings will provide guidance for reframing the clinical perspective regarding SPC treatments for patients with hypertension taking multiple pills. Addressing the questions listed above will also better guide the clinical evaluation and care of patients with hypertension. However, clinical practices are still facing technical challenges involved in SPC regimens, especially the dosages, formulations, combinations, administration schedule, and workmanship.

## CONFLICTS OF INTEREST

The authors declare no potential conflicts of interest with respect to the research, authorship, and/or publication of this manuscript.

## AUTHORS CONTRIBUTIONS

Y. Li, and M. Narisawa wrote the first draft of the manuscript. Z. Huang, X. Meng, H. Wang, X. Jin, and X. Shen edited the manuscript. X.W. Cheng handled the funding and supervision.
